# Implementation evaluation of the Dutch national heat plan among long-term care institutions in Amsterdam: a cross-sectional study

**DOI:** 10.1186/1472-6963-13-135

**Published:** 2013-04-11

**Authors:** Anton E Kunst, Rieneke Britstra

**Affiliations:** 1 Department of Public Health, Academic Medical Center (AMC), University of Amsterdam, PO Box 22660, Amsterdam, 1100 DD, The Netherlands

**Keywords:** Heatwaves, Elderly, Institutions, Prevention, Nursing care

## Abstract

**Background:**

In 2007, a national heat plan was introduced in the Netherlands to effectively protect vulnerable populations (such as institutionalised elderly people) against heatwaves. The aim of this study was to assess the extent to which the measures recommended in this heat plan had been implemented, and could be implemented, in long-term care institutions in Amsterdam three years on.

**Methods:**

Questionnaires were sent to the care managers of all 54 eligible long-term care institutions in Amsterdam. This included questions on the presence of a heat protocol and cooling facilities in the building. Furthermore, the care managers were asked to judge the importance of 23 of the cooling measures recommended by the National Heat Plan in the event of a heatwave, and to report on practical problems that may affect the implementation of these cooling measures.

**Results:**

Of the 54 questionnaires sent, 27 were returned. Most institutions had a heat protocol, virtually all of which had been developed in the three years preceding the survey. Outdoor sunshades were used most often to protect residents against heat (93% of all institutions). Prevalence of cooling facilities such as air conditioning and rooftop cooling had increased, but remained low (41%). Care managers confirmed the importance of most of the 23 cooling measures recommended by the National Heat Plan, with some exceptions. Only 41% regarded consulting physicians on medication use to be ‘very important’. Most care managers did not foresee large problems with the implementation of the recommended cooling measures. Barriers mentioned related to shortage of and expertise among personnel, and residents’ independence.

**Conclusion:**

The results suggest that a national heat plan could be implemented in long-term care institutions with few problems. Possible areas of improvement include cooling of buildings and staff training.

## Background

Heatwaves in 2003 and 2006 resulted in large increases in mortality rates in the Netherlands [1,2]. Heat-related mortality may become an increasingly important problem, as global warming will increase the future frequency and intensity of heatwaves [3-7]. Different climate scenarios predict average global temperature levels to increase over the coming decades [3]. Scenarios for the Netherlands’ central region predict that the annual number of days with temperatures above 25 degrees Celsius will increase from 24 days previous to the year 2000 to between 30 and 47 days in 2050 [4]. Moreover, the ageing of the population, which may be fuelled by sustained increases in life expectancy over the coming decades [8], will result in an increase in the number of people aged 80 and above.

A large number of studies have identified population groups that are at increased risk of dying during heatwaves [5-7]. An important risk group is elderly people living in institutions such as homes for the elderly, nursing homes, and other long-term care facilities [9-16]. In the Netherlands as well as in other countries, the institutionalised elderly face increased mortality risks during heatwaves. This increase is larger than among elderly people who do not live in institutions. For example, in the Netherlands in 1993–94, the relative risk of dying during periods of high outdoor temperatures (25°C–29.9°C) was 1.50 for nursing home patients compared to 1.09 for the entire Dutch elderly population [17]. In Southern England in 2003, mortality rates during heatwaves increased by 50% among nursing home residents under the age of 75, and by 72% among residents of homes for the elderly, compared to a 13% increase among elderly people living at home [12]. During heatwaves in Italy in 2003, mortality rates increased by 50% in nursing homes, while in France, mortality rates increased by 100% in retirement homes [11,13].

After the 2006 heatwaves, the Dutch Ministry of Health, in collaboration with various partners, prepared a national heat plan aimed at protecting vulnerable groups against the health hazards of future heatwaves [2]. The National Heat Plan was officially implemented in July 2007. The plan identified several risk groups: elderly people in general, people with chronic illnesses, obese people, socially isolated people, and residents of institutions. For the latter, the Plan recommends cooling measures both at the level of institutions (such as use of sunshades and ventilation) and at the level of individual residents (such as intensified care and behavioural adaptations).

In 2008, during a summer with no heatwaves, an internet-based survey was used among nurses to assess the implementation of the National Heat Plan [18]. Of the nurses who responded, 34% reported they would take the initiative to use cooling measures if outdoor temperatures reached high levels. Another 35% of the nurses reported they would take such measures only if the residents requested them, while 31% would not take any measures at all. Since 2008, there have been no new evaluations of the implementation of the National Heat Plan.

This study aimed to evaluate in more detail the implementation of the heat plan within institutions for the elderly. We assessed the extent to which the measures recommended in the 2007 heat plan had been implemented, and could be implemented, in institutions in Amsterdam three years on. The evaluation included three elements. First, we assessed which cooling facilities were present in the institutions’ buildings in 2010, and whether the prevalence of these facilities had increased since 2007. Second, we assessed whether the care managers of these institutions acknowledged the importance of 23 of the cooling measures recommended by the National Heat Plan. Third, we assessed which practical problems were foreseen with regard to the implementation of the recommended measures in the event of a heatwave.

## Methods

All eligible institutions within the boundaries of the city of Amsterdam were approached for participation. In total, we identified 54 institutions, including nursing homes, homes for the elderly (i.e. residential institutions where elderly occupants have their permanent address), facilities that combine nursing homes and homes for the elderly, and a few other types of facilities (e.g. houses with community living arrangements). We did not include hospices, hospitals, or offices of home care providers where no residents lived. We restricted the study to Amsterdam, as it was carried out in collaboration with the Public Health Service of Amsterdam.

Eligible institutions were invited to participate by means of an introductory letter accompanied by a hard copy of the questionnaire. The letter was addressed to the central care manager of each institution – more specifically, to the person responsible for organising, coordinating, and supervising the care delivered in the institution at large. To non-respondents, we sent emails with electronic copies of the questionnaire three and five weeks after sending the original questionnaire. The survey was conducted between 30 April and early June 2010 during a period of temperate weather.

The questionnaire contained questions on general characteristics of the institution, the presence of a heat protocol, and the presence of cooling facilities in the building. Furthermore, the providers were asked to judge the importance of 23 of the cooling measures the National Heat Plan recommends should be taken during heatwaves, and to answer questions on practical problems that may affect the implementation of these measures. The selection of the cooling facilities and the 23 cooling measures was derived from the National Heat Plan and from a related manual by ActiZ, the national organisation of care institutions [2,19]. The 23 measures were selected from a longer list of about 40 different measures recommended in these documents. The selected measures were representative with regard to type of measure and degree of effort required.

Of these 23 cooling measures, 11 were measures taken at the level of the entire institution (e.g. use of sunshades, ventilation, electronic equipment, and sprinkling water on terraces and rooftops), while 12 were measures for individual residents (e.g. adjustments in daily schedule, intensity of activities and therapies, extra fluid supply, physical care and protection during sun exposure). For each measure, the respondent was asked to rate its importance as a cooling measure during a heatwave. Answer categories were ‘less important’, ‘important’, and ‘very important’. In addition, for each measure, the respondent had to indicate the extent to which problems could be expected during the implementation of this measure. Answer categories were ‘no problems’, ‘some problems’, and ‘large problems’.

The questionnaire was piloted among the care managers of two institutions outside the study area. This pilot resulted in only a few minor changes to the questionnaire.

The answers to the questionnaire were processed using the programme Microsoft Excel. Computations consisted of frequency counts for individual variables or for combinations of variables.

The study did not need to be approved by an ethics committee in the Netherlands.

## Results

Twenty-seven (50%) of the 54 delivered questionnaires were returned. These questionnaires represent nine nursing homes (33%), six homes for the elderly (22%), five combinations of the previous two (19%), six assisted living facilities (22%), and one other type of institution (4%).

Care managers of 24 institutions answered questions regarding the presence of a heat protocol. A heat protocol was present in 16 of these 24 institutions (= 67%). In 15 of these 16 institutions, the heat protocol was established within the 3 years preceding the survey. In three more institutions, a heat protocol was in development at the time of the survey. In the remaining five institutions (= 21%), there was no heat protocol present or in development at the time of the survey.

Table 1 lists the prevalence of cooling facilities within specific parts of the buildings. In 25 (= 93%) of all 27 institutions, outdoor sunshades were available to protect the living rooms of all residents. Outdoor sunshades were available in 89% of all institutions to protect common rooms (e.g. living rooms, libraries, or recreation rooms), in 85% to protect outdoor terraces, and in 83% to protect their restaurants and cafés. Only a few of these facilities were installed between 2007 and 2010.

**Table 1:** Prevalence (as percentage of all responding institutions) of cooling facilities at different sites within the institution

**Cooling facilities**	**Site**		***Prevalence***^***[*a*]***^		***Constructed in the past 3 years***^[a]^	
Outdoor sunshade	Central lobby	61%	(14/23)	0%	(0/11)
	Common rooms^b^	89%	(24/27)	0%	(0/19)
	Restaurant/café	83%	(20/24)	6%	(1/16)
	Terrace	85%	(22/26)	11%	(2/19)
	Residents’ rooms/apartments	93%	(25/27)	5%	(1/20)
Air conditioning	Central lobby	5%	(1/22)	0%	(0/1)
	Common rooms	19%	(5/27)	60%	(3/5)
	Restaurant/café	17%	(4/23)	50%	(2/4)
	Reception	4%	(1/24)	0%	(0/1)
	Residents’ rooms/apartments	8%	(2/25)	50%	(1/2)
Ceiling fans	Central lobby	4%	(1/25)	n.a.	(0/0)
	Common rooms	19%	(5/26)	40%	(2/5)
	Restaurant/café	22%	(5/23)	0%	(0/3)
	Reception	4%	(1/25)	n.a.	(0/0)
	Residents’ rooms/apartments	7%	(2/27)	50%	(1/2)

^a^Between brackets: positive cases/total number of institutions. The latter number may be lower than 27 due to non-response or because the cases are not applicable.

^b^Rooms accessible to all residents, including living rooms, libraries, and recreation rooms.

Air conditioning and ceiling fans were much less common than outdoor sunshades. Air conditioning was most prevalent in the common rooms (19%) and restaurants and cafés (17%). Although air conditioning and fan prevalence had increased since 2007, their prevalence remained low in 2010.

Several institutions reported other technical facilities to regulate the climate inside their buildings (results not shown). Rooftop insulation was present in 44% of the institutions, while 30% reported having high-yield (i.e. heat-reflective) window glass. There had been a slight increase in these cooling facilities between 2007 and 2010. Of all institutions, 22% had two or more cooling facilities other than sunshades, 37% had one such facility, while 41% reported having no cooling facilities other than sunshades (results not shown).

Table 2 shows to what extent the care managers acknowledged the importance of the cooling measures recommended for the institution at large. The majority of the care managers acknowledged that most of these measures were ‘important’ or ‘very important’. There was much consensus on the importance of lowering the sunshades already at sunrise (‘very important’ for 44% of the care managers). On the other hand, some cooling measures were regarded as ‘less important’ by at least one-third of the care managers, including turning off heat-producing equipment, switching off lighting, sprinkling water on rooftops and terraces, and having a cooled room accessible to all residents.

**Table 2:** **Percentage of care managers who agreed on the importance and feasibility of institutional-level measures, by cooling measure**^**a**^

**Institutional-level cooling measure**	**Importance**			**Feasibility**		
	***Less important***	***Important***	***Very important***	***No problems***	***Some problems***	***Large problems***
a) Lowering sunshade between 12.00 and 16.00	4%	30%	67%	72%	24%	4%
b) Lowering sunshade already at sunrise	26%	30%	44%	64%	36%	0%
c) In the evening and at night: natural or mechanical ventilation	0%	67%	33%	32%	48%	20%
d) Closing the windows when outside temperature exceeds inside temperature	8%	50%	42%	64%	16%	20%
e) Turning off heat-producing equipment (e.g. television) when possible	56%	44%	0%	32%	40%	28%
f) Sparing lighting in case of heat distribution	33%	52%	15%	54%	33%	13%
g) Sprinkling water on the rooftops and terraces	37%	52%	11%	33%	25%	42%
h) A central cooled room accessible to all residents	35%	46%	19%	29%	17%	54%
i) Closing curtains in case of invading sunlight	11%	59%	30%	56%	28%	16%
j) Air conditioning switched on during part of the day	18%	45%	36%	42%	11%	47%
k) Switching on fans during part of the day	0%	77%	23%	61%	26%	13%

^a^Because of rounding-off errors, some percentages add up to 99% or 101%.

Table 3 shows the extent to which care managers acknowledged the importance of the cooling measures recommended for individual residents. In general, in-dividual-level measures were more often regarded to be ‘important’ or ‘very important’ than measures at the level of the institution at large (see Table 2). An extra round of beverages with active offering of fluids was most often regarded as ‘important’ (89%), followed by the passive offering of fluids during the day (78%). However, adjusting the daily schedule of residents was considered to be ‘less important’ by a significant minority of the care managers (38%). Perhaps most notably, consulting a physician when residents are taking medication was considered ‘very important’ by less than half of the care managers (41%).

**Table 3:** **Percentage of care managers who agreed on the importance and feasibility of individual-level measures, by cooling measure**^**a**^

**Individual-level cooling measure**	**Importance**			**Feasibility**		
	***Less important***	***Important***	***Very important***	***No problems***	***Some problems***	***Large problems***
a) Offering passive fluids by placing water jugs during the day	0%	22%	78%	85%	8%	8%
b) Extra round of drinks with active offering of fluids, soup or juices	0%	11%	89%	81%	12%	8%
c) Avoiding sun exposure during 12.00 and 16.00	0%	33%	67%	81%	19%	0%
d) Stimulating wearing loose clothing and help changing if necessary	0%	48%	52%	58%	38%	4%
e) Stimulating and helping residents to move to cooled rooms	8%	50%	42%	8%	75%	17%
f) Adjusting daily schedule: waking up early and keeping siesta	38%	46%	15%	31%	58%	12%
g) Reducing frequency and intensity of activities and therapies	7%	70%	22%	69%	31%	0%
h) Stimulating and helping residents to splash their face, neck and wrists	7%	74%	19%	62%	38%	0%
i) Washing ill residents upon need, possibly by disposable bed bath	4%	63%	33%	81%	19%	0%
j) Consulting a physician when residents are taking medication	15%	44%	41%	77%	23%	0%
k) Stimulating active use of sunshades among residents	22%	48%	30%	62%	35%	4%
l) Stimulating covering the head during sun exposure, using a hat or cap	0%	41%	59%	69%	27%	4%

^a^Because of rounding-off errors, some percentages add up to 99% or 101%.

When asked about problems that were expected to occur when implementing the recommended cooling measures, 22 of the 27 care managers mentioned 1 or more problems (results not shown). In total, 47 problems were listed. Of these, 17 related to practical or technical problems, such as substandard physical conditions of the building and the absence of required technical facilities. A further 13 problems related to personnel, including personnel shortage, negligence, and lack of knowledge. Finally, the care managers made 17 references to problems related to the residents, including their lack of awareness of risks (4 items) and the need to respect their independence and personal responsibility (11 items).

The presence of a heat protocol in an institution was not consistently associated with the perceived importance of cooling measures mentioned in the National Heat Plan. Care managers whose institution had a heat protocol considered institutional-level measures to be ‘very important’ slightly more often (32% of all answers compared to 23% for other managers). However, no such difference was observed for individual-level measures (48% versus 55%).

## Discussion

As early as 1997, a Dutch study concluded that ‘more should be done to prevent nursing home patients from dying during periods with high outdoor temperatures’ and that ‘…introduction of climate control (air conditioning) should be seriously considered’ [17], p. 1298]. However, a national heat plan was not implemented until July 2007, in the aftermath of serious heatwaves in 2003 and 2006. Particular attention was given to the protection of elderly residents in institutions like nursing homes, where much of the heat-related mortality occurred.

In Amsterdam, after three years of implementation, the balance of benefits was mixed. Protection of elderly residents against outdoor heat still depended largely on the use of outdoor sunshades. There had been a slight increase in additional cooling facilities such as air conditioning, but such additional facilities were still absent in nearly half of the institutions. In the event of a heatwave, most institutions had a heat protocol specifying which cooling measures should be taken. Virtually all of these protocols were developed in the three years preceding the survey. Care managers generally agreed on the importance of cooling measures recommended by the National Heat Plan, and most of them foresaw few problems with their implementation. Nonetheless, not all measures were acknowledged as equally important, and some managers expected serious barriers related to personnel or residents.

### Evaluation of data

The response rate to the survey was 50%. The sample included different types of institutions, with the number of residents varying from 10 to 400. However, a comparison with published figures for the Netherlands [20] suggests that nursing homes were over-represented in our sample. When care managers were contacted with reminders, they reported lack of time as the main reason for not returning the questionnaire. Non-response might be higher among institutions with less interest in the development and implementation of heat protocols. If so, the implementation of heat protocols in Amsterdam is less advanced than our results suggest.

We carefully checked the response patterns for indications of response bias, such as inaccurate or sloppy reporting, or a tendency to give socially desirable answers. For example, regarding the 11 questions on cooling measures for the institution at large (Table 2), two care managers rated all items as ‘important’, while two other care managers reported that they expected no problems with the implementation of the cooling measures. However, the response patterns of all other care managers were more varied, and many care managers provided extensive replies to the open question on the implementation problems they expected.

By carrying out a survey in the spring, we deliberately measured the extent to which institutions were prepared to cope with heatwaves before they could actually occur. As a result, we did not assess the measures that were actually implemented during the heatwaves that occurred in July 2010, nor did we assess the problems that were encountered in practice. However, anecdotal evidence on the 2010 heatwaves supports the care managers’ expectations about the main implementation problems. Newspaper articles in July 2010 reported the key problems to be shortage of personnel, lack of expertise among employed personnel (e.g. on the most effective cooling measures), and the need to respect the residents’ independence and personal responsibility.

The situation in Amsterdam may not be representative of other cities and regions in the Netherlands. One reason is that the frequency and intensity of heatwaves differs greatly across the country in relationship to the degree of urbanisation and proximity to the sea [4]. Similarly, the results may not be generalisable to other countries. The level of protection against heat waves may vary between countries in relationship to climatic factors, the level of investment in institutional care, and the composition of the resident population. In the Netherlands, elderly people are institutionalised only if they are highly dependent on care. Domiciliary care is increasingly being developed for elderly people with a lesser degree of dependency [21]. Only about 5000 persons (less than 1% of the total population of Amsterdam) live in the institutions covered by our study [22]. In institutions that host less dependent residents it may perhaps be easier to implement heat protocols, e.g. by granting greater autonomy to residents.

### Interpretation and implications

The number of cooling facilities increased only slightly during the three years following the implementation of the National Heat Plan in 2007. This slow increase may be understandable, given the large investments required to buy and install many cooling measures (such as air conditioning), particularly in older buildings. Unfortunately, this slow rate of investment does not ensure rapid progress in preventing heat-related mortality. A faster rate may be achieved by focussing on less expensive facilities such as rooftop cooling and heat-reflective window glass.

According to the National Heat Plan of 2007 [2], a large number of cooling measures can be taken when heatwaves occur. While these recommendations were said to be effective [2], some of them were regarded to be ‘less important’ by a substantial minority of the care managers. It could be that the National Heat Plan had not been sufficiently disseminated among institutions. However, we found that the care managers’ assessment of the importance of cooling measures was unrelated to the presence of a heat protocol in their institutions. This lack of a relationship might reflect poor dissemination of heat plans *within* institutions. Furthermore, the perceived effectiveness of some recommended measures may strongly depend on local conditions. For example, depending on the type of building, sprinkling water on rooftops and terraces may be feasible and effective in some places but not in others.

One out of seven care managers considered consulting a physician when residents are taking medication to be ‘less important’. Several studies have demonstrated an increased risk of heat-related mortality among patients who take specific types of medication, such as diuretics [23] and long-term antihypertensive medication [24]. National heat plans and local protocols should place greater emphasis on patients who take specific types of medication, and raise greater awareness among health care professionals on the risks involved for these people.

Some implementation problems relate to the shortage of and expertise among personnel. It is important to assess how the alertness and performance of personnel can be enhanced during heatwaves by specific measures, including modifications to work schedules and information on health risks. Another implementation problem relates to the need to respect residents’ responsibility and self-determination. Nonetheless, for confused, disoriented, or disabled residents, health care providers have a particular responsibility to protect them against their own actions or lack thereof. Moreover, especially those residents with the greatest mental and physical disabilities are at increased risk of death during heatwaves [17].

In this study, heat plan implementation was assessed from the perspective of health care managers. As in surveys among health officials [13], the results may not represent the views and experiences of front-line staff. Though the responding care managers endorsed most of the measures recommended in the National Heat Plan, support may be lower among front-line staff. For example, in a Dutch internet survey, only one-third of the responding nurses said they would take measures proactively if outdoor temperatures reached high levels [18]. Further studies among front-line staff are needed to assess whether heat protocols really make a difference to residents of institutions.

## Conclusions

Three years after the National Heat Plan was implemented, most of the responding institutions had developed a heat protocol. Care managers recognise the importance of the cooling measures recommended by the National Heat Plan, and foresee few problems with their implementation. Further development and implementation of national heat plans and local heat protocols is essential to protect the institutionalised elderly against future heatwaves. Potential areas of improvement include the cooling of buildings and training of staff. Future studies should assess the cost-effectiveness of measures aimed at preventing heat-related morbidity and mortality among the institutionalised elderly. Moreover, implementation studies in other countries would provide a broader evidence base to support the implementation of heat protocols in different contexts.

## Competing interests

The authors declare that they have no competing interests.

## Authors’ contributions

AK conceived the idea for this study. RB carried out the survey fieldwork, and data analysis, and was advised by AK. AK and RB wrote the paper jointly. Both authors read and approved the final version of the manuscript.

## Pre-publication history

The pre-publication history for this paper can be accessed here:

http://www.biomedcentral.com/1472-6963/13/135/prepub

## References

[B1] Garssen J Harmsen C de Beer J The effect of the summer 2003 heat wave on mortality in the Netherlands Euro Surveill 2005 10 165 168 16088044

[B2] Ministerie van Volksgezondheid Nationaal hitteplan 2007 The Hague, The Netherlands; Min VSW

[B3] Intergovernmental Panel on Climate Change (IPCC) Core Writing Team, Pachauri RK, Reisinger A Climate change 2007: synthesis report. Contribution of working groups I, II and III to the fourth assessment report of the intergovernmental panel on climate change 2007 Geneva, Switzerland: IPCC

[B4] Royal Dutch Meteorological Institute (KNMI) Climate change scenarios 2006 for the Netherlands. KNMI report WR2006-01 2006 De Bilt, The Netherlands: KNMI

[B5] Aström DO Bertil F Joacim R Heat wave impact on morbidity and mortality in the elderly population: a review of recent studies Maturitas 2011 69 99 105 10.1016/j.maturitas.2011.03.008 21477954

[B6] Gasparrini A Armstrong B The impact of heat waves on mortality Epidemiology 2011 22 68 73 10.1097/EDE.0b013e3181fdcd99 21150355 PMC3324776

[B7] Kovats RS Shakoor H Heat stress and public health: a critical review Annu Rev Public Health 2008 29 41 55 10.1146/annurev.publhealth.29.020907.090843 18031221

[B8] Janssen F Kunst A The choice among past trends as a basis for the prediction of future trends in old-age mortality Popul Stud 2007 61 315 326 10.1080/00324720701571632 17979005

[B9] Klenk J Becker C Rapp K Heat-related mortality in residents of nursing homes Age and Ageing 2010 39 245 252 10.1093/ageing/afp248 20093248

[B10] Hajat S Kovats RS Lachowycz K Heat-related and cold-related deaths in England and Wales: who is at risk? Occup Environ Med 2007 64 93 100 16990293 10.1136/oem.2006.029017 PMC2078436

[B11] Rozzini R Zanetti E Trabucchi M Elevated temperature and nursing home mortality during the 2003 European heat wave J Am Med Dir Assoc 2004 5 138 139 15015220

[B12] Kovats RS Johnson H Griffith C Mortality in southern England during the 2003 heat wave by place of death Health Stat Q 2006 29 6 8 16523675

[B13] Matthies F Menne B Prevention and management of health hazards related to heatwaves Int J Circumpolar Health 2009 68 8 22 19331238 10.3402/ijch.v68i1.18293

[B14] Kovats RS Kristie LE Heatwaves and public health in Europe Eur J Public Health 2006 16 592 599 10.1093/eurpub/ckl049 16644927

[B15] Borst V Schols JMGA Mackenbach JP Toegenomen sterfte van verpleeghuispatiënten bij extreme buitentemperatuur; toename groter bij hitte dan bij koude Ned Tijdschr Geneeskd 1997 141 2180 2183

[B16] Kovats RS Hajat S Heat stress and public health: a critical review Annu Rev Public Health 2008 29 41 55 10.1146/annurev.publhealth.29.020907.090843 18031221

[B17] Mackenbach JP Borst V Schols JM Heat-related mortality among nursing-home patients Lancet 1997 349 1297 1298 9142072 10.1016/S0140-6736(05)62510-X

[B18] Beroepsvereniging van zorgprofessionals Hitte kan gevaarlijk zijn! Neem maatregelen http://www.venvn.nl/Actueel/Nieuwsarchief/tabid/1789/Articleid/567/mid/3452/Default.aspx

[B19] Actiz Houdt het hoofd koel: aandachtspunten bij hoge temperaturen 2007 Utrecht, The Netherlands; Actiz

[B20] RIVM Aantal verpleeg- en verzorgingshuizen per gemeente 2009 http://www.zorgatlas.nl/zorg/langdurige-zorg/verpleging-en-verzorging/

[B21] CBS Statline http://statline.cbs.nl/StatWeb/dome/?LA=EN

[B22] Mateboer-Bos M Swinkels HAM van Hilten O, Voorrips LE, Boerdam AA Ontwikkelingen zorggebruik ouderen Gezondheid en zorg in cijfers 2012 2012 The Hague: Statistics Netherlands (CBS) 97 118

[B23] Faunt JD Wilkinson TJ Aplin P Henschke P Webb M Penhall RK The effecte in the heat: heat-related hospital presentations during a ten day heat wave Australian and New Zealand Journal of Medicine 1995 25 117 121 10.1111/j.1445-5994.1995.tb02822.x 7605292

[B24] Arguad L Ferry T Le QH Marfisi A Ciorba D Achache P Duclazeau R Robert D Short- and long-term outcomes of heatstroke following the 2003 heat wave in Lyon, France Arch Intern Med 2007 167 E1 E7 10.1001/archinte.167.20.ioi70147 17698677

